# Transcriptome-wide analysis supports environmental adaptations of two *Pinus pinaster* populations from contrasting habitats

**DOI:** 10.1186/s12864-015-2177-x

**Published:** 2015-11-06

**Authors:** Rafael A. Cañas, Isabel Feito, José Francisco Fuente-Maqueda, Concepción Ávila, Juan Majada, Francisco M. Cánovas

**Affiliations:** Departamento de Biología Molecular y Bioquímica, Facultad de Ciencias, Instituto Andaluz de Biotecnología, Universidad de Málaga, Campus Universitario de Teatinos s/n, 29071 Málaga, Spain; Sección Forestal, SERIDA, Finca Experimental La Mata, 33825 Grado, Principado de Asturias Spain

**Keywords:** Adaptation, Light signalling, Oligonucleotide microarray, *P. pinaster*, RNA-Seq, Provenances, Water use

## Abstract

**Background:**

Maritime pine (*Pinus pinaster* Aiton) grows in a range of different climates in the southwestern Mediterranean region and the existence of a variety of latitudinal ecotypes or provenances is well established. In this study, we have conducted a deep analysis of the transcriptome in needles from two *P. pinaster* provenances, Leiria (Portugal) and Tamrabta (Morocco), which were grown in northern Spain under the same conditions.

**Results:**

An oligonucleotide microarray (PINARRAY3) and RNA-Seq were used for whole-transcriptome analyses, and we found that 90.95 % of the data were concordant between the two platforms. Furthermore, the two methods identified very similar percentages of differentially expressed genes with values of 5.5 % for PINARRAY3 and 5.7 % for RNA-Seq. In total, 6,023 transcripts were shared and 88 differentially expressed genes overlapped in the two platforms. Among the differentially expressed genes, all transport related genes except aquaporins were expressed at higher levels in Tamrabta than in Leiria. In contrast, genes involved in secondary metabolism were expressed at higher levels in Tamrabta, and photosynthesis-related genes were expressed more highly in Leiria. The genes involved in light sensing in plants were well represented in the differentially expressed groups of genes. In addition, increased levels of hormones such as abscisic acid, gibberellins, jasmonic and salicylic acid were observed in Leiria.

**Conclusions:**

Both transcriptome platforms have proven to be useful resources, showing complementary and reliable results. The results presented here highlight the different abilities of the two maritime pine populations to sense environmental conditions and reveal one type of regulation that can be ascribed to different genetic and epigenetic backgrounds.

**Electronic supplementary material:**

The online version of this article (doi:10.1186/s12864-015-2177-x) contains supplementary material, which is available to authorized users.

## Background

Conifers are the most abundant group of gymnosperms and include tree species of great ecological and economical relevance. Conifers cover vast areas within the forest of the northern hemisphere and are extensively exploited as the main source of wood for industrial purposes [1].

The abundance of polysaccharides, pigments and phenolic compounds in the lignified tissues of conifers has hampered research on the molecular biology of these plants for many years. Until the development of recent adapted protocols, RNA [2] and DNA [3] extractions from conifer tissues were difficult and extremely laborious. However, in the last decade considerable progress has been made in establishing adapted protocols and techniques that are now utilized in molecular studies of conifer biology. These include methods such as laser-capture microdissection [4, 5], microarray hybridization [6] and both stable [7, 8] and transient transformation [9, 10].

Although conifer genomes are extremely large, ranging from 18 to 35 gigabases, recent developments in next-generation sequencing have facilitated new advances in genomics research [11, 12]. In the past few years the assembly of several conifer transcriptomes [13–15] and genomes has been reported [16–18]. Despite these recent developments, the use of genome-wide expression techniques based on sequencing (RNA-Seq) in conifer species has remained limited [19–21]. Furthermore, the lack of reference genome/transcriptome for read mapping generally necessitates the use of self-assemblies of the transcriptomes.

These new genomic tools have enormous potential for use in studies of the molecular basis of genetic diversity and the adaptations of conifers to the environment. This is important because trees with a wide geographical distribution have a variety of latitudinal and altitudinal ecotypes (provenances) adapted to local light and climate conditions [22]. A certain inherent flexibility in the response to varying temperature and photoperiod conditions has been observed in trees [22]. One such example is the well-studied response of conifers to extreme winter periods when they are subjected to cold, drought and oxidative stresses [23]. Conifers have developed a series of responses to these environmental changes including the accumulation of “compatible solutes” [24–27], adjustments of membrane lipid composition [28] and changes in energy metabolism that prioritize oxidative phosphorylation when photosynthesis is arrested [25, 27, 29–31].

Because of its economic and environmental potential, the maritime pine (*Pinus pinaster* L. Aiton) is one the most important conifer species in the southwestern Mediterranean region. Maritime pine is also a conifer species with advanced genomic research in Europe and a large number of genomic resources have been generated in the last few years [5, 15, 27, 32].

This pine species has high phenotypic plasticity with high tolerance to abiotic stresses such as drought and is widely distributed in different environments and climates [33–35]. Intraspecific variability has been observed in hormone production between *P. pinaster* populations growing under different environmental conditions [24, 33]. Therefore, the maritime pine is an excellent model for studying the molecular basis of environmental adaptation using functional genomic approaches.

Transcriptome profiling has previously been conducted in conifers to explore the molecular basis of phenotypic changes [6, 36]. In this study, a large-scale custom microarray (4x44k) containing 60-mer oligonucleotide probes (PINARRAY3) was designed to represent the *P. pinaster* transcriptome [15]. In parallel, RNA-Seq was performed using a subset of 35,374 contigs from SustainPineDB as a reference [15]. The platforms have been compared, showing complementary and reliable results. Microarray and RNA-seq were used to explore transcriptome changes in phenotypically divergent *P. pinaster* populations grown under the same conditions. A transcriptome-wide analysis was conducted in the needles of two maritime pine provenances from contrasting habitats, Leiria and Tamrabta, phenotypically divergent and with distinct potential of environmental adaptation. We hypothesize that phenotypic divergence is supported, at least in part, by changes in the transcriptome. The results presented in this study highlight the adaptive responses of the two provenances as a result of their different abilities to sense the environmental conditions, thereby suggesting regulation by different genetic and/or epigenetic backgrounds.

## Results

### Transcriptomic analyses

In this study, we have developed new technical resources for functional genomics analysis of *Pinus pinaster*. A new oligonucleotide microarray (60-mer) called PINARRAY3, synthesized by Agilent (Santa Clara, CA, USA), has been developed to study the transcriptome of maritime pine. This array contains 45,220 spots including negative and positive controls (2,720) and sequences for unique transcripts (42,500) in the maritime pine transcriptome included in the SustainPineDB v3.0 [15]. To increase specificity, the 3’UTR of the transcripts (when available) was used in the design of the 60-mer oligonucleotides. This microarray has been tested with RNA samples from adult needles of two geographically distinct populations of maritime pine, Leiria and Tamrabta, which were chosen for their contrasted habitats and diverse phenotypes (Additional file 1: Figures S1 and S2). The Leiria provenance is from central Portugal, in the European Atlantic region, and Tamrabta is from the Moroccan Atlas Mountains (Additional file 1: Figure S2). Trees of both provenances were cultivated in the same monitored fields in northern Spain where Leiria showed higher rates of growth (54.7 cm/year) and survival rate (91.7 % in two years) than Tamrabta (41.2 cm/year and 76.7 % in two years) and also displayed more heterogeneity.

**Fig. 1:**
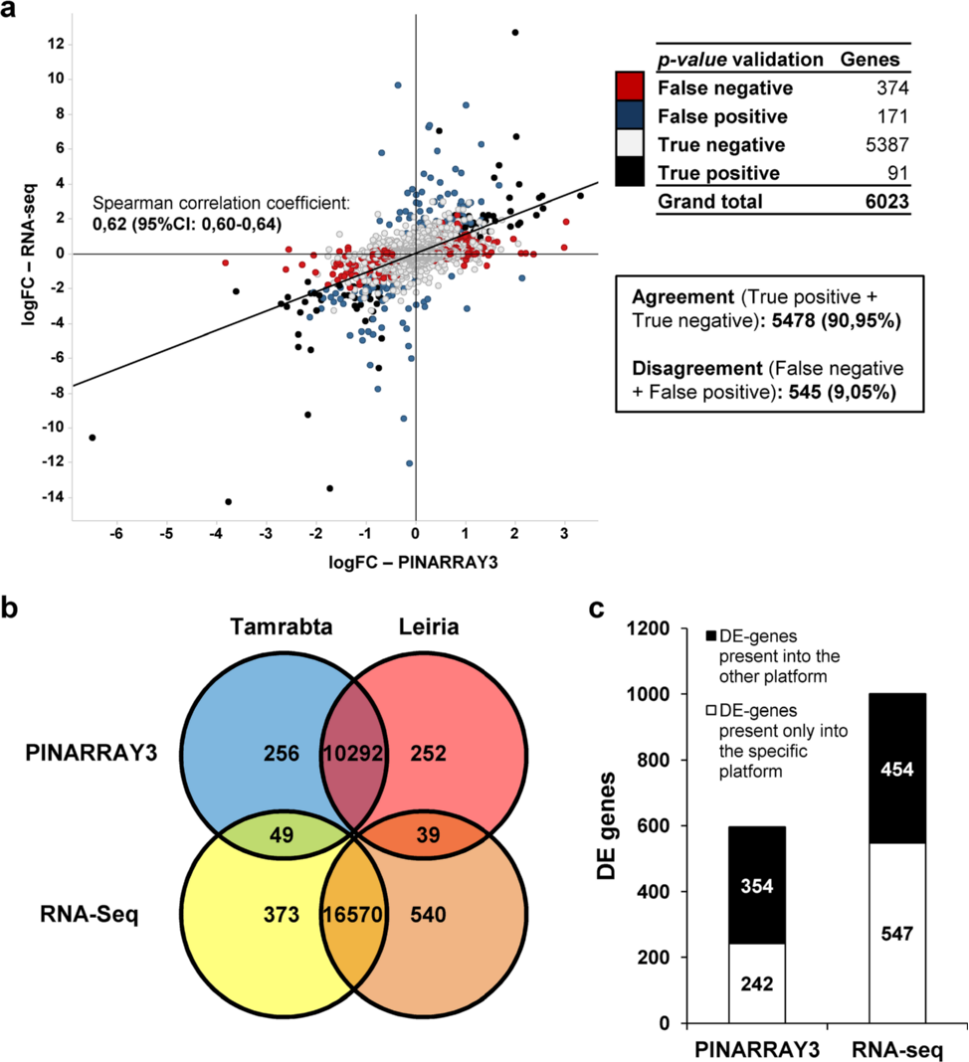
Comparative analysis of PINARRAY3 and RNA-Seq. **a** Comparison of fold changes (logFC) between PINARRAY3 and RNA-Seq analyses. Red dots represent ‘False negative’ genes (significant for PINARRAY3 and not significant for RNA-Seq). Blue dots represent ‘False positive’ genes (not significant for PINARRAY3 and significant for RNA-Seq). Open dots represent ‘True negative’ genes (not significant for PINARRAY3 and RNA-Seq). Black dots represent ‘True positive’ genes (significant for PINARRAY3 and RNA-Seq). The trend line is adjusted to a Spearman correlation. **b** Venn diagram showing the results for the DE analysis from both PINARRAY3 and RNA-Seq. **c** DE genes by platform indicating their inclusion in one or both platforms

Eight microarray fields were hybridized with 4 biological replicates from each provenance in an alternating manner. The raw microarray data were normalized and analyzed using the Limma R package [37] resulting in 10,888 spots with a signal that was significantly different from that of the background (Fig. 1b, Additional file 2: Table S1). Of these, 596 spots were considered to represent differentially expressed (DE) genes (logFC > 0.5; *adjP.value* < 0.05), with 305 showing higher expression in Tamrabta (negative values of logFC) and 291 showing higher expression in Leiria (positive values of logFC) (Fig. 1b, Additional file 2: Table S1). In the samples analyzed there were higher inter-provenance than intra-provenance variations between microarray results in a multidimensional scaling plot (Additional file 1: Figure S3). However, Tamrabta showed higher variability than Leiria likely reflecting the effect of the environment.

RNA-Seq was chosen as the second method of analysis in this transcriptome study. Two of the RNA samples hybridized with PINARRAY3 were sequenced. A subset of 35,374 contigs in the SustainPineDB [15] was used as reference transcriptome sequence for read mapping. In total, 17,571 genes were represented, and 1001 genes showed differential expression between the provenances (logFC > 1; FDR < 0.05, Fig. 1b, Additional file 2: Table S2). A higher logFC value was used to consider a gene as differential expressed in the RNA-Seq because of the lower number of replicates (*n* = 2). There were 422 genes more highly expressed in Tamrabta (negative values of logFC) whereas 579 genes showed higher expression levels in Leiria (positive values of logFC). The results are presented in Fig. 1b and Additional file 2: Table S2.

To evaluate the reproducibility and validity of the transcriptomic data obtained, results from both methods were compared using Spotfire software. From the expressed transcripts in PINARRAY3 and RNA-Seq only 6,023 were shared between both platforms and used for the comparison (Fig. 1a, Additional file 2: Table S3). The logFC values of these genes had a Spearman correlation coefficient of 0.62 (Fig. 1a). The Spotfire software only used the *adjP.value* (<0.05) or FDR (<0.05) values to divide genes into four categories: ‘False negative’, comprising 374 genes (significant for PINARRAY3 and not significant for RNA-Seq); ‘false positive’, comprising 171 genes (not significant for PINARRAY3 and significant for RNA-Seq); ‘true negative’, comprising 5387 genes (not significant for PINARRAY3 and RNA-Seq); and ‘true positive’, comprising 91 genes (significant for PINARRAY3 and RNA-Seq). Of the total data compared, 90.95 % were concordant between the PINARRAY3 and RNA-Seq (True positives and True negatives) (Fig. 1a). When the DE genes in both platforms were compared with a more restrictive criterion including logFC values (PINARRAY3, logFC > 0.5; *adjP.value* < 0.05. RNA-Seq, logFC > 1, FDR < 0.05), 49 were found to be overexpressed in Tamrabta and 39 were found to be overexpressed in Leiria (Fig. 1b).

To determine the DE genes in each platform, only 6,023 genes could be used for the comparison. Among the 596 DE genes identified using PINARRAY3, 354 genes were differentially expressed according to both analyses and 242 only showed differential expression using PINARRAY3 (Fig. 1c). Moreover, of the 1001 DE genes identified using RNA-Seq, 454 were differentially expressed according to both analyses, and 547 were only differentially expressed according to RNA-Seq (Fig. 1c).

**Fig. 2:**
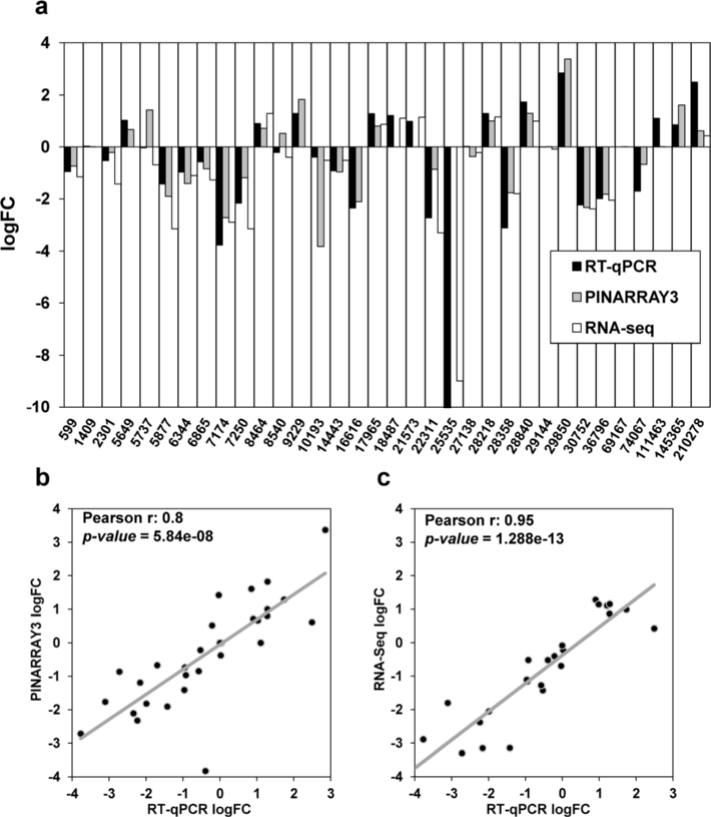
Validation of PINARRAY3 and RNA-Seq analyses by qPCR. **a** Fold changes (logFC) of gene expression in Leiria and Tamrabta provenance samples analyzed using PINARRAY3, RNA-Seq and qPCR are shown. Each gene is identified by its numeric ID in the database SustainPine v3.0. Positive values correspond to higher expression in Leiria samples and negative values to higher expression in Tamrabta samples. **b** Pearson correlation between logFC values determined from PINARRAY3 and qPCR analysis. **c** Pearson correlation between logFC as determined using RNA-Seq and qPCR analyses

Both methods were validated using qPCR analysis (Fig. 2) of 36 genes, including *Actin* and *EF1A*. In general, the logFC results obtained via the three methods were similar except for a few cases such as the sp_v3.0_unigene8540 or the sp_v3.0_unigene5737, for which the PINARRAY3 data differed from the qPCR and RNA-Seq data (Fig. 2a). The Pearson correlation between qPCR and PINARRAY3 logFC was significant (*P* = 5.84e-08), with a *r* index of 0.8 (Fig. 2b), and the correlation between qPCR and RNA-Seq logFC was significant (*P* = 1.288e-13), with a *r* index of 0.95 (Fig. 2c).

### Functional enrichment analysis

**Fig. 3:**
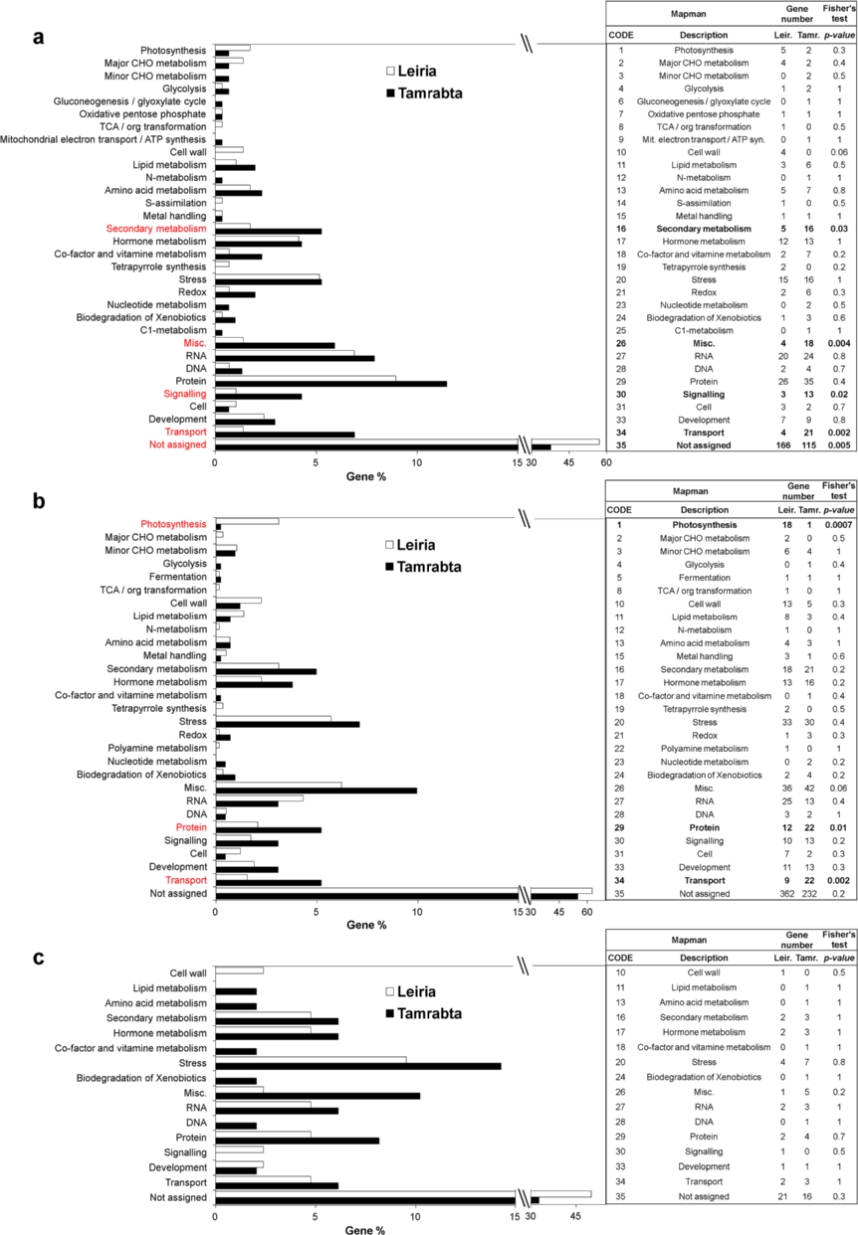
Global functional enrichment analysis of the PINARRAY3 and RNA-Seq results. The horizontal bars represent the percentage of genes included in each functional category. The functional enrichment analysis was based on the functional Mapman categories and performed using the Fisher’s exact test. Significant categories have *P-values <* 0.05. The significant categories are shown in red in graphs and in bold in tables. **a** Functional enrichment analysis for PINARRAY3 results. **b** Functional enrichment analysis for RNA-Seq results. **c** Functional enrichment analysis for significant genes in both PINARRAY3 and RNA-Seq

**Fig. 4:**
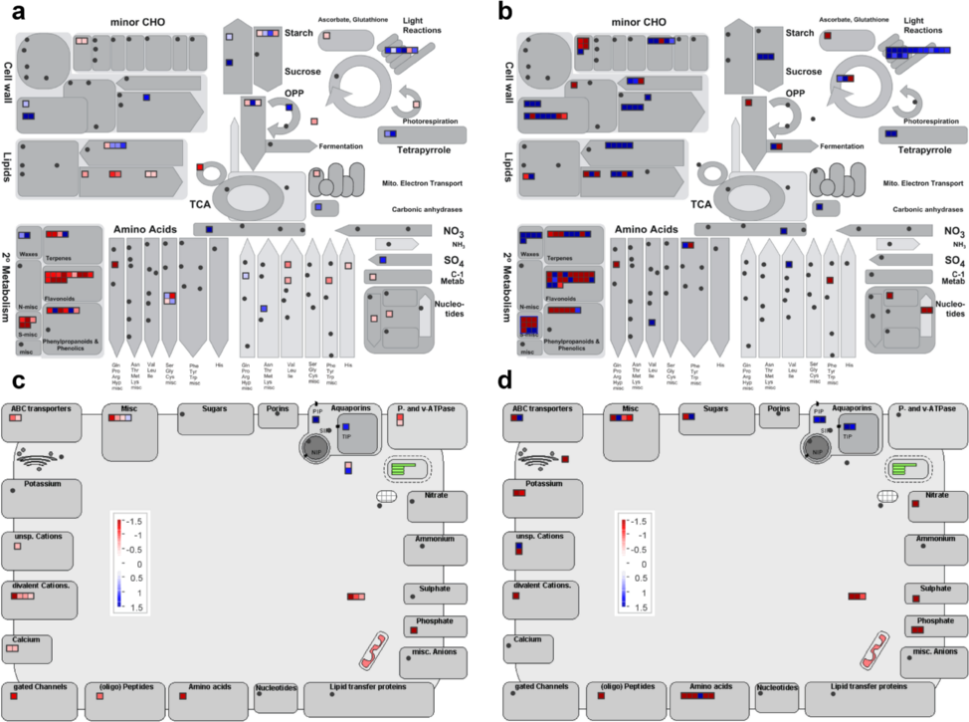
Mapman diagram of the PINARRAY3 and RNA-Seq results. The results of the differentially expressed genes in the PINARRAY3 and RNA-Seq analyses are presented. Red boxes represent genes with higher expression in Tamrabta samples and blue boxes represent genes with higher expression in Leiria samples. The expression of the genes involved in “metabolism” category is presented in the panels as follows: **a** PINARRAY3 and **b** RNA-Seq. The expression of genes belonging to the enriched functional category “transport” is presented in the panels as follows: **c** PINARRAY3 and **d** RNA-Seq

Gene function enrichment analysis was performed using the Mapman categories through the sequence annotation file obtained using the Mercator web tool [38]. The enrichment analysis results are shown in Figs. 3 and 4 and in Additional file 2: Table S4. Of the 35 Mapman categories, 32 were represented in the DE genes identified using PINARRAY3, 29 were found among the DE genes identified using RNA-Seq and only 16 fell into the group of common DE genes (Fig. 3). In all cases the most abundant category was “unassigned genes” (Fig. 3). There were 5 categories with significant differences between both provenances as determined using PINARRAY3 (Fisher’s exact test, *P-value <* 0.05): secondary metabolism (16), miscellaneous (26), signaling (30), transport (34) and unassigned genes (35) (Fig. 3a). RNA-Seq analysis yielded 3 categories showing significant differences in a Fisher’s exact test: photosynthesis (1), protein (29) and transport (34) (Fig. 3b). None of the categories were significantly different in the common results (Fig. 3c). Except for the category of unassigned genes (35) in PINARRAY3 and photosynthesis (1) in RNA-Seq, where Leiria showed more significant genes, the rest of significant categories had more significant genes in Tamrabta (Fig. 3). In the remaining categories, the differences between provenances were due to the expression of different genes and not to the number of DE genes (Additional file 2: Table S4), e.g. hormone metabolism (17), RNA (27), protein (29) metabolism and signaling (30).

To further examine the DE genes involved in metabolism and transport, the results were represented in Mapman (Fig. 4). The secondary metabolism genes (phenylpropanoids, flavonoids and isoprenoids) were enhanced in the Tamrabta provenance compared to the Leiria provenance and genes involved in carbon metabolism (cell wall, lipid biosynthesis, glycolysis, starch metabolism and photosynthesis) were upregulated in Leiria (Fig. 4a and b). When depicted using the KEGG metabolic maps, the higher expression in Tamrabta of genes from the oxidative phosphorylation pathway was especially prominent (Additional file 1: Figure S4). In addition, the UDP-glycosyltransferases were differentially expressed in Tamrabta (Additional file 2: Table S4). With the exception of aquaporins, which were more abundant in Leiria than in Tamrabta, transport-related genes were more abundant in Tamrabta (Fig. 4b and c).

**Fig. 5:**
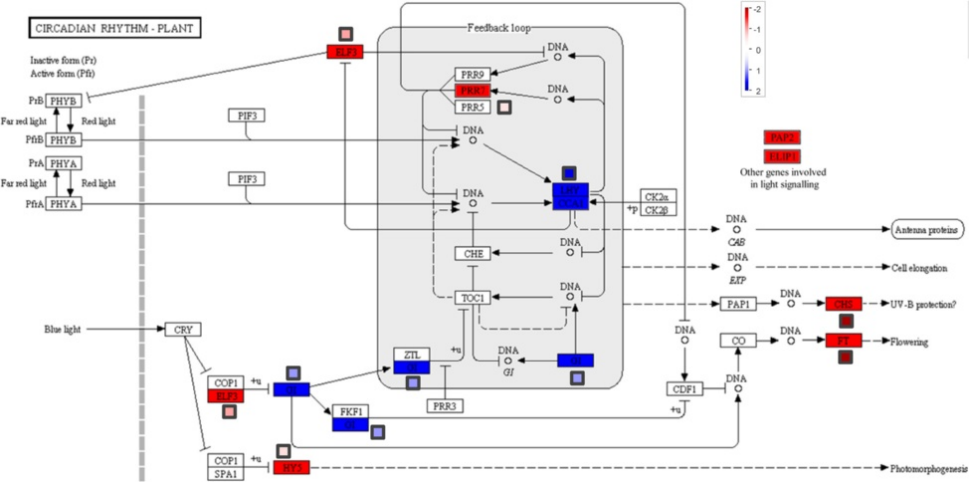
KEGG representation of the plant circadian rhythm including DE genes. Schematic view of the regulation pathway of the plant circadian rhythm including DE genes from PINARRAY3 and RNA-Seq. Red boxes indicate genes with higher expression in Leiria samples and green lines indicate genes with higher expression in Tamrabta samples. Mapman boxes show the logFC results from the qPCR analysis

Based on the PINARRAY3 and RNA-Seq analyses, genes involved in light sensing or circadian rhythms were well represented in the group of DE genes. Although the orthologues of *GIGANTEA* (sp_v3.0_unigene5649) and *LHY/CCA* (sp_v3.0_unigene28840) were expressed at higher levels in Leiria, the rest of the genes involved in these processes were expressed more highly in Tamrabta than in Leiria (Fig. 5). Examples include *PRR7* (sp_v3.0_unigene2301), *ELF3* (sp_v3.0_unigene6344), *HY5* (sp_v3.0_unigene6865), *FT* (sp_v3.0_unigene22311), chalcone synthase (sp_v3.0_unigene30752), *ELIP* (sp_v3.0_unigene97042) and *PAP2* (sp_v3.0_unigene37920). The qPCR and transcriptomic expression data for this group of genes were consistent, as shown in Fig. 5.

### Levels of secondary metabolites and hormones

**Fig. 6:**
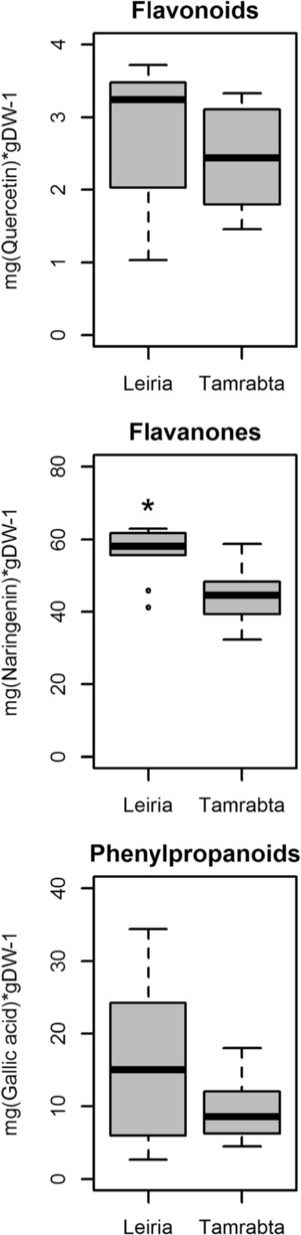
Flavonoids and phenylpropanoids in the needles of Leiria and Tamrabta. Boxplots show the levels of total flavonoids, flavanones and phenylpropanoids in needles from the Leiria and Tamrabta provenances. Significant differences were calculated using Student’s *t*-test. Significant differences are shown with a single asterisk for *P-values <* 0.05

The differential expression of genes coding for enzymes involved in secondary metabolism prompted us to determine the relative levels of flavonoids and phenylpropanoids in the two maritime pine provenances (Fig. 6) using two different methods previously reported [39, 40]. Although the traditional AlCl_3_ method cannot detect flavanones, it is used for quantification of total flavonoids. The 2,4-dinitrophenylhydrazine colorimetric method measures flavanones independently of other of flavonoids [40]. There were no significant differences in flavonoid and phenylpropanoid accumulation between Leiria and Tamrabta, although the amounts were slightly higher in Leiria (Fig. 6). However, there was a significant difference in the accumulation of flavanones, with significantly higher levels present in Leiria than in Tamrabta (Fig. 6).

**Fig. 7:**
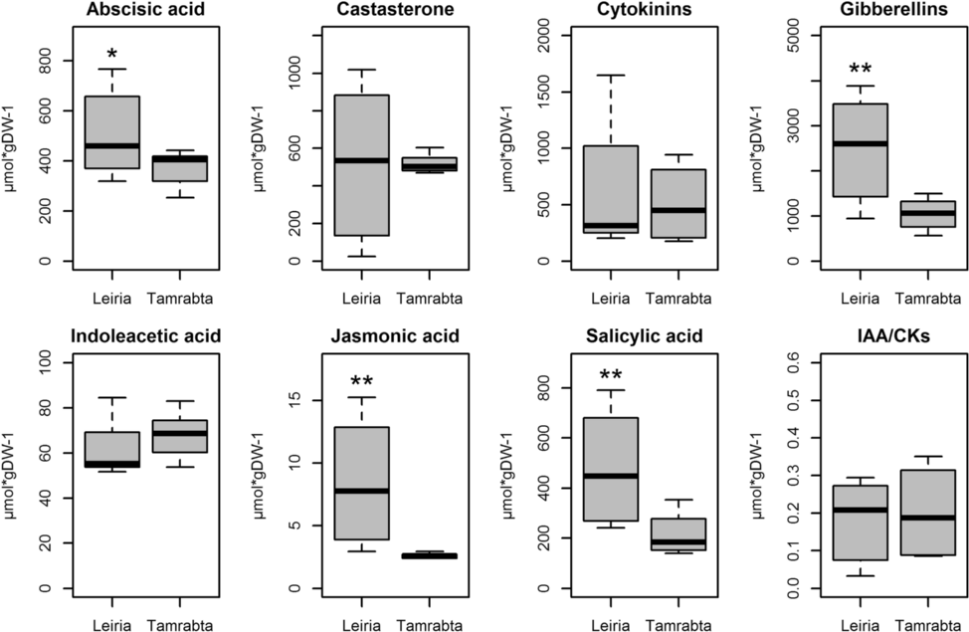
Hormones in the needles of Leiria and Tamrabta. Boxplots show the levels of asbcisic acid (ABA), castosterone, cytokinins, gibberellins (GAs), indoleacetic acid (IA), jasmonic acid (JA) and salicylic acid (SA) and the indoleacetic acid:cytokinins index in needles from the Leiria and Tamrabta provenances. Significant differences were calculated using Student’s *t*-test. Significant differences are shown with a single asterisk for *P-values <* 0.05 and two asterisks for *P-values <* 0.01

Because the hormone metabolism category included a high number of DE genes (25 genes in PINARRAY3 and 29 in RNA-Seq) (Fig. 3), hormone levels were determined in the needles of Leiria and Tamrabta. This included measurement of abscisic acid, castasterone, cytokinins, gibberellins, indoleacetic acid, jasmonic acid, salicylic acid and an evaluation of the relationship between indoleacetic acid and cytokinins. Significant differences were observed for abscisic acid (ABA), gibberellins (GAs), jasmonic acid (JA) and salicylic acid (SA), and the levels were always higher in Leiria than in Tamrabta (Fig. 7).

## Discussion

### Comparison of PINARRAY3 and RNA-Seq data

In this study, we used two different approaches for transcriptome profiling in maritime pine (*Pinus pinaster*): microarray (PINARRAY3) analysis and RNA-Seq. These two approaches were based on the *de novo* assembly of transcriptome data available at SustainPineDB, the *P. pinaster* gene expression database [15], and proved to be powerful tools for producing reliable functional results with a good correlation (Fig. 1). The comparison of microarray and RNA-Seq data with data obtained from qPCR analysis showed very strong correlations, validating the power of both techniques’ analysis of gene expression on a genome-wide scale. The qPCR data support the differential expression of genes that were only identified in PINARRAY3 and also of genes that were only identified in RNA-Seq (Fig. 5). All these data indicate that the selected genes were not false positives and further suggest a low number of false positives in our transcriptomic analyses. However, RNA-Seq presented better correlations with qPCR than did PINARRAY3 (Fig. 2). This may indicate technical limitations of microarray analysis due to the use of nucleotide hybridization, which can introduce biases such as cross-hybridization of multiple labeled target transcripts to the same probe, increasing the rate of false positives and diminishing the quantification precision in regard to RNA-Seq. Despite the low number of biological replicates (2) in the RNA-Seq compared to microarray method (4), the reliability of the results supports further use of RNA-Seq [41].

RNA-Seq and microarray analysis have been proposed to be complementary methods for transcriptome profiling [42]. Although PINARRAY3 and the RNA-Seq reference transcriptomes represent approximately 90 % of the protein-coding genes of maritime pine and both were selected from the same database (SustainPineDB), the methods for contig selection were different resulting in different sequence pools as reflected in Fig. 1. This is due to the lack of complete and reliable gene annotation in conifers, although the sequencing and assembly of three genomes have recently been published [16–18]. The lack of a reference genome hinders the implementation of sequencing-based techniques, such as RNA-seq, in genome-wide analyses and supports the use of de novo assemblies for RNA-seq studies [19–21]. Nevertheless, SustainPineDB has yielded good results, thereby validating the database construction and making the microarray and RNA-Seq data more comparable.

### Analysis of the Leiria and Tamrabta transcriptomes

PINARRAY3 and RNA-Seq were used to compare transcriptomes from the needles of two different *P. pinaster* provenances, Leiria and Tamrabta, which have different geographical origins and phenotypes (Additional file 1: Figure S1 and S2). *P. pinaster* is a forest tree species with a fragmented distribution that limits gene flow between populations and likely increase the genetic divergence between ecotypes [43–45]. However, a recent study about resistance to cavitation in *P. pinaster* has shown that genetic and phenotypic differentiation for certain traits are limited [46]. Leiria and Tamrabta were grown in the same place under the same environmental conditions and, consequently, few differences in gene expression were observed between the two provenances (Fig. 1). The percentage of DE genes was very similar in the two platforms used (5.5 % for PINARRAY3 and 5.7 % for RNA-Seq), confirming the reliability of both genomic tools for transcriptome analysis in maritime pine samples

The analysis of functional categories revealed differences in adaptation responses to environmental conditions between the two maritime pine provenances. In general, metabolite transport appeared to be increased in Tamrabta. However, the increased expression of aquaporins (*AQP*) in Leiria suggest significant differences in the management of water and CO_2_ (Fig. 4). It has been observed that *AQP*-overexpressing plants have higher photosynthetic rates and faster growth [47, 48]. Consistent with these observations, Leiria grew taller and showed increased expression of photosynthesis-related genes compared to Tamrabta (Figs. 3 and 4, Additional file 1: Figure S1). Phenologically, Leiria matures earlier than Tamrabta, especially in unfavorable conditions such as cold weather. However, under optimal conditions Tamrabta completes its growth cycle more rapidly. To compensate for its weaker adaptive capacity, Tamrabta has evolved to complete the growth cycle during periods of favorable light and temperature. In contrast, Leiria, which is more suited to Atlantic conditions, grows for a longer period and reaches a greater height. However, the higher GA content in Leiria correlates with increased elongation rather than an increase in the number of whorls.

The precedent findings suggest that Leiria tends to use more water (water expender) and that Tamrabta uses less (water saver). Water saver plants reduce gas conductance and transpiration by maintaining constant minimum relative water content and leaf water potential under conditions of water stress. This results in decreased photosynthesis and lower growth rates. In contrast, water expender plants always maintain a high evaporative demand, allowing a decrease in leaf water content during drought to maintain high photosynthesis and growth rates [49]. Unsurprisingly, the water saver strategy is typical of plants from arid zones, and plants without water supply problems usually adopt the water expender behavior. Moreover, Leiria demonstrated no down-regulation of AQP gene expression in response to ABA and accumulated higher levels of AQP, which is an additional characteristic of water expender plants (Figs. 4 and 7). This suggests that even drought-tolerant species can have different intraspecific responses to water stress. It has been observed that *Pinus radiata* (*Pinaceae*) has an ABA-driven stomatal closure during water stress, which is a typical water saving behavior. In contrast, in *Callitris rhomboidea* (*Cupressaceae*), stomatal closure is induced by increased ABA levels, which decline with prolonged water stress. Therefore, the stomatal closure in this species is driven by water potential, which is a water-expender behavior [50]. Because Leiria and Tamrabta were grown under the same conditions, the differential response observed may be explained by the high plasticity of the response of *P. pinaster* to environmental conditions, and this response would possibly be under genetic and epigenetic control.

Consistent with the argument above, the DE gene and functional enrichment analyses also suggest that the adaptations provide different capacity to sense environmental conditions. Therefore, the differential expression of several genes involved in light signaling may result from this phenomenon (Fig. 5, Additional file 2: Table S4). Moreover, the differences in the accumulation of hormones such as ABA, JA and SA (Fig. 7) that are involved in UV-B responses also support this hypothesis [51]. The finding of different hormone levels between provenances is according with previous works about JA accumulation in *P. pinaster* [35]. Because the latitudes of the maritime pine provenances studied here are very different, their responses to photoperiod and light intensity must also be different. Among conifer populations from different latitudes, an allele frequency cline and divergent expression patterns of *FTL2*, a member of the *TERMINAL FLOWER 1* gene family involved in the photoperiod sensing, has been observed in Norway spruce and Scots pine [52, 53]. In the present study, one member of the *TERMINALFLOWER 1* family, *FLT1* (sp_v3.0_unigene22311), was overexpressed in Tamrabta (Fig. 5, Additional file 2: Table S4). Based on our results, the higher expression of the conifer *LHY-CCA1* homolog gene (sp_v3.0_unigene28840) in Leiria may indicate different adaptation to light conditions between provenances (Fig. 5). It is known that *LHY* (AT1G01060) and *CCA1* (AT2G46830) are transcription factors involved in the circadian clock and cold acclimation in *Arabidopsis* [54]. However, in *Picea abies* the unique conifer *LHY/CCA1* homolog (KC311521) showed diurnal expression cycles but no strong circadian clock, with rapid dampening in free-running conditions [55]. Because the epigenetic control of these signaling routes is well known [22], it is conceivable that the differences observed between Leiria and Tamrabta may be partially due to epigenetic responses since this work has been made with clonal propagated individuals grown in a greenhouse under the same environmental conditions.

An additional indication of the higher adaptive capacity of Leiria to varying growth conditions is the profile of DE genes involved in lipid metabolism. In Tamrabta, genes encoding enzymes directly involved in the oxidative phosphorylation and membrane proteins involved in the ATP/energy metabolism associated to oxidative phosphorylation were overexpressed (Fig. 4, Additional file 1: Figure S4, Additional file 2: Table S4). This type of response is induced during winter when the energy produced by photosynthesis decreases, necessitating new sources of energy [25, 27, 29–31]. In addition, the levels of the growth hormones GA were lower in Tamrabta (Fig. 7), and GA synthesis in plants is known to be inhibited by cold [56]. Taken together, these results suggest that southern provenances may respond more slowly to photoperiod as previously observed in Norway spruce and Scots pine [53].

Consistent with these findings the accumulation of flavanones (a type of flavonoids) was higher in Leiria than in Tamrabta (Fig. 6). In plants flavonoid amount and profile can change due to UV-B stress mediated by hormones such as ABA, JA and SA [51]. However, several genes involved in secondary metabolism, mainly in the biosynthesis of flavonoids, were expressed at higher levels in Tamrabta than in Leiria (Figs. 3 and 4, Additional file 2: Table S4). These results suggest that the difference in flavonoid profiles between Tamrabta and Leiria is due to genetic and/or epigenetic differences in their response to environmental stimuli. Accordingly, the miscellaneous category of genes (26) contains an important representation of genes for UDP-glycosyltransferases and glutathione-S-transferases, which are more expressed in the Tamrabta provenance (Fig. 3, Additional file 2: Table S4). UDP-glycosyltransferases are key enzymes that dictate differences in the structure of flavonoids [57]. Modifications in flavonoid structure and composition may constitute a chemical fingerprint for the *P. pinaster* varieties or provenances.

## Conclusions

Whole-transcriptome resources are now accessible for non-classical model organisms. In this study, oligonucleotide microarrays and RNA-Seq analyses were used to characterize the maritime pine (*Pinus pinaster)* transcriptome, for which they produced reliable results. Transcriptome profiling of needle samples from two maritime pine provenances validated both genomic platforms and elucidated the genetic basis of the differences in environmental adaptation between them. The observed differences in gene expression between Leiria and Tamrabta supported the hypothesis that genetic and epigenetic factors of the provenances play a significant role in the modulation of specific responses to environmental conditions.

## Methods

### Plant material

In this study, two conifer provenances of contrasting phenotypes and from different geographical distributions have been used as xeric and mesic models (Additional file 1: Figures S1 and S2). Leiria, a Portuguese provenance (Leiria; 39° 27’ 36” N, 08° 34’ 48” W; 55 m), grows near the coast with warm winters and exhibits high plasticity in biomass allocation under water stress treatment [58, 59]. Tamrabta, from the Atlas mountains in Morocco (Tamrabta; 33° 36’ 00” N, 05° 01’ 12” W; 1,758 m), exhibits high allocation to roots, low allocation to stem and low growth potential. The Tamrabta population grows at a higher altitude than Leiria with a short growing season and where access to soil water may be more important than efficient water use [33]. These provenances are included in the clonal collection CLONAPIN [60] and were propagated by cuttings from progenies previously tested in field trials. Rooted plant cuttings 17 month-old were planted in 2 L pots filled with 4:1 (by volume) mixture of Sphagnum peat (PINSTRUD®) and grade 3 vermiculite (VERLITE®). The entire experiment was performed at SERIDA’s greenhouse at Villaviciosa (Asturias, Spain). Potted plants were ferti-irrigated following the protocol in [60]. Prior to the assessment, all branches were pruned to trigger the formation of new axillary shoots as a result of activation of the proventitious meristems in the extant dwarf shoots. This was intended to be a “resetting” of the plant shoots to minimize the differences due to pruning previously performed for cutting collection. All samples were harvested the same day at the same time. The harvested needles were immediately frozen in liquid N and subsequently placed in dry ice for the transport to the laboratory. All frozen samples were reduced to a homogenous powder with a mills mixer MM400 (Retsh, Haan, Germany) and stored at −80 °C until further use for metabolite and RNA extractions.

### Development of an oligonucleotide microarray for maritime pine (PINARRAY3)

A custom gene expression microarray (PINARRAY3) was designed using the Agilent Technologies eArrayH web application. Probe sequences were obtained using GE Probe Design considering 3’ end biased 60-mer oligonucleotides and using the *P. pinaster* transcriptome available in SustainPineDB v3.0 as the reference [15].

The microarrays including the standard panel of quality control and spike-in probes available from Agilent Technologies were manufactured using Agilent SurePrintTM Technology in the 4x44 format with 42,500 spots from SustainPineDB v3.0 contigs. The PINARRAY3 platform has been deposited in the NCBI’s Gene Expression Omnibus [61] and is accessible through GEO Series with the accession number GPL19234 (http://www.ncbi.nlm.nih.gov/geo/query/acc.cgi?acc=GPL19234).

### RNA extraction

Total RNA was isolated following the protocol described in [62] and modified in [9]. The RNA concentration and purity were determined via spectrophotometry using A260/A280 nm and A260/A230 nm ratios. RNA quality was further confirmed using agarose gel electrophoresis and using the RNA Pico Assay for the 2100 Bioanalyzer (Agilent, USA). RNA samples with an RNA integrity number (RIN) higher than 7 were used for RNA-Seq. The amount of the RNA for RNA-Seq was calculated using the Quant-iT™ RiboGreen® RNA Assay Kit (Invitrogen, Paisley, UK).

### cRNA synthesis, sample labeling and microarray hybridization

In total, 8 samples were amplified and labeled for microarray hybridization; 4 for each provenance used in this study. The samples L6, L7, L8 and L9 were prepared from Leiria, and samples T3, T6, T7 and T8 were prepared from Tamrabta. The cRNA synthesis and labeling were performed using the Low Input Quick Amp Labelling Kit, One-Color (Agilent Technologies) following the manufacturer’s ‘One-Color Microarray-Based Gene Expression Analysis’ protocol. cRNA synthesized from 200 ng of total RNA was labeled with Cyanine 3-CTP. Each PINARRAY3 field was hybridized with 1.65 μg of labeled cRNA. After labeling, the samples were purified using the NucleoSpin® Gel and PCR Clean-up kit (Macherey-Nagel, Düren, Germany). Dye incorporation and cRNA yield were estimated via spectrophotometry using a NanoDrop ND-1000.

Microarray hybridization was performed in a SureHyb microarray hybridization chamber with gasket slides (Agilent Technologies) at 65 °C in a rotary oven for 17 h. After hybridization, microarrays were washed with GE Wash Buffer 1 (Agilent) at room temperature for 1 min, GE Wash buffer 2 (Agilent) at 37 °C for 1 min, acetonitrile for 10 s and Agilent’s stabilization and drying solution for 30 s and, then air dried. Hybridized slides were scanned at 5 μm resolution and their signal intensities were detected using a GenePix 4100A microarray scanner (Molecular devices, Sunnyvale, CA, USA). The raw data preprocessing, quantile normalization and analysis of differentially expressed genes were performed with the Limma package for R [37] (Additional file 1: Note S1). Bayes statistics were applied to determine the differential expression between the comparisons. Statistical significance was corrected for multiple testing using the Benjamini-Hochberg procedure. A multidimensional scaling plot for samples was made using the *plotMDS* function of Limma with default parameters (Additional file 1: Figure S3). The microarray data have been deposited in the NCBI’s Gene Expression Omnibus [61] and are accessible through GEO Series with the accession number GSE61801 (http://www.ncbi.nlm.nih.gov/geo/query/acc.cgi?acc=GSE61801).

### RNA-Seq

RNA sequencing was conducted in the Centro Nacional de Análisis Genómico (CNAG, Barcelona, Spain) sequencing facility. RNA libraries for sequencing were prepared using TruSeq RNA kits (Illumina, San Diego, CA, USA) according to the manufacturer’s instructions. Clustering was performed on a cBot cluster generation system using an Illumina HiSeq paired-end read cluster generation kit according to the manufacturer’s instructions (Illumina, San Diego, CA, USA). The 4 samples (T3 and T6 from Tamrabta provenance, L6 and L7 from Leiria provenance) were sequenced on an Illumina HiSEq 2000 as paired-end reads of 100 bp. All lanes were spiked with a 1–2 % phiX control library. The sequencing runs were performed according to the manufacturer’s instructions. Base conversion was performed using Illumina’s OLB version 1.9. A total of 113.7; 137.2; 125.3 and 190.6 millions of reads were obtained for the T3, T6, L6 and L7 samples, respectively.

The resulting reads were trimmed and analyzed using SeqTrimNext software [63]. Only the pairs in which both reads passed the quality test were further analyzed. After read trimming, a total of 110.4, 128.7, 121 and 180.5 millions of paired-end reads were recovered for the T3, T6, L6 and L7 samples, respectively. These reads were mapped onto a reference transcriptome designed from the SustainPine Database (http://www.scbi.uma.es/sustainpinedb/home_page). This reference transcriptome is composed of 35,374 contigs (Additional file 2: Table S5) and was constructed based on the annotation results of the Full Lengther Next (FLN) software (http://rubydoc.info/gems/full_lengther_next/0.0.8/frames). The read mapping was performed with Bowtie2 [64] allowing only for concordant mapped pairs (−no-mixed–no-discordant). Read count was performed with a Phyton script from the Andalusian Platform of Bioinformatics. Differentially expressed genes were identified using the edgeR package for R, Additional file 1: Note 2 [65]. The PINARRAY3 and RNA-Seq results were compared using the Spotfire software (TIBCO, Boston, MA, USA). The RNA-Seq data have been deposited in the NCBI’s Gene Expression Omnibus [61] and are accessible through GEO Series with the accession number GSE61923 (http://www.ncbi.nlm.nih.gov/geo/query/acc.cgi?acc=GSE61923).

### Functional gene enrichment analyses

The mapping files for the Mapman representation were made using the Mercator web server [38], and the Mapman images were made using the Mapman software [66]. The gene enrichment comparison analyses between the two provenances were performed using Fisher’s exact tests in R environment (http://www.R-project.org) with the Mapman functional categories. The differences between provenances were considered significant with *P <* 0.05. Ortholog assignment and pathway mapping of significant genes were made with the KEGG Automatic Annotation Server (KAAS) [67] using the SBH option and the gene dataset as described in Additional file 1: Note S3.

### qPCR analyses

Validation of the gene expression results obtained via microarray was performed using qPCR. Total RNA (500 ng) was used to synthesize cDNA using the iScript™ Reverse Transcription Supermix for qPCR (BioRad, CA, USA). Following the instruction manual the reverse transcription conditions were: 5 min at 25 °C, 30 min at 42 °C and finally 5 min at 85 °C. The reverse transcription was carried out in a thermal cycler DNA Engine® (BioRad, CA, USA). The qPCR reactions were carried out with 5 ng of cDNA per reaction as described in [5]. The primers used for the qPCR reactions are listed in Additional file 2: Table S6. The raw fluorescence data from each reaction was fitted to the MAK2 model, which requires no assumptions about the amplification efficiency qPCR assay [68]. The initial target concentrations (D0 parameter) for each gene was deduced from the MAK2 model using the qpcR package for the R environment [69] and normalized to the geometric mean of two reference genes, *Actin* and *EF1A*. In total, 4 biological and 3 technical replicates were made for each sample analyzed using qPCR. Pearson correlations were performed using the R environment (http://www.R-project.org).

### Total phenol, flavonoid and flavanone determination

Total phenol and flavonoid analysis was carried out following the modified protocol in [70]. Plant needles were collected, immediately immersed in liquid nitrogen, lyophilized (Telstar LyoQuest, Terrassa, Spain) and powdered (6770 Freezer/Mill® Cryogenic Grinder, Thomas Scientific, NJ, USA). The samples (1 g) were extracted in 30 mL of methanol at 25 °C, for 1 h in an overhead shaker (Reax 2, Heidolph Instrument, Schwabach, Germany) and filtered through Whatman No. 4 paper. The residue was then extracted adding an additional 30 mL portion of methanol. The combined methanol extracts were evaporated at 35 °C under vacuum conditions (Laborota 4002, Heidolph Instrument, Schwabach, Germany), re-dissolved in methanol at a concentration of 10 mg/mL and stored at 4 °C for future use.

Total phenolic content was determined by the Folin and Ciocalteu reagent method (Compendium of International Methods of Analysis, FolinCiocalteu Index, OIV-MA-AS2-10), adapted for a 96-well plate assay. Aliquots of the methanolic extract solutions (10 μL) were used, oxidized with the Folin-Ciocalteu reagent (15 μL) and the reactions neutralized with 60 μL of sodium carbonate 20 % (p/v). Final reaction volume was made up to 300 μL, with distilled water. Absorbance was measured at 750 nm (Synergy HT, Biotek Instrument, Vertmon, USA) after 30 min at 25 °C. The calibration curve was performed with gallic acid, and the results expressed as mg of gallic acid equivalent (GAE) per g of dry weight.

The total flavonoid content was determined using the method described in [39], adapted for a 96-well plate assay. 100 μL of 2 % aluminiumtrichloride in methanol was mixed with the same volume of plant extract. The absorbance reading at 415 nm (Synergy HT, Biotek Instrument, Vertmon, USA) was taken after 10 min against a blank sample consisting of 100 μL of sample solution and 100 μL of methanol. The total flavonoid content was determined using a standard curve of quercetin at 0–50 mg/L. The average of three readings was used and the results expressed as mg of quercetin equivalent (QE) per g of dry weight.

Flavanones were extracted from 100 mg powder needle sample in 1.5 mL ethanol 95 % over night at 4 °C under continuous shake (200 rpm). The debris was eliminated by centrifugation at 25,000 xg for 15 min at 4 °C. The supernatant can be stored at −20 °C until use. Flavanones were measured using the 2,4-dinitrophenylhydrazine colorimetric method described in [40] with some modifications. To 30 μL of each sample were sequentially added 60 μL of 1 % 2,4-dinitrophenylhydrazine reagent (1 g of 2,4-dinitrophenylhydrazine dissolved into 2 mL of 96 % sulfuric acid and then diluted to 100 mL with methanol) and 60 μL of methanol. The mix was incubated at 50 °C for 50 min. After cooling to room temperature the absorbance was measured at 495 nm. (±)-Naringenin was used as the reference standard. The total flavonoid content was determined using a standard curve of (±)-naringenin at 0–4000 mg/L. The average of three readings was used and the results expressed as mg of (±)-naringenin equivalent (NE) per g of dry weight.

Boxplots and Student’s *t* test were made using R environment (http://www.R-project.org). Differences were considered significant with a *P-value <* 0.05.

### Hormone measurements

Plant material was collected, frozen in liquid nitrogen and stored until extraction at −80 °C. Samples were freeze dried (Telstar LyoQuest, Terrassa, Spain). 60 mg of lyophilized plant needles were ground into powder (Fast Prep. FP 120, Qbiogene Inc.,CA, USA). The analysis of different plant growth regulators (epibrasinolide, 24 EB; abscisic acid, ABA; indolacetic acid, AIA; benziladenine, BA; castasterone, BK; dihydrozeatin, DHZ; dihydrozeatinriboside, DHRZ; gibberellins GA1, GA3, GA4, GA7 and GA9; homobrasinolide, HBI; isopentenyladenine, IP; isopentenyl adenosine, IPR; jasmonicacdi, JA; salicylic acid, SA; zeatin, Z and zeatinriboside, RZ) was carried out using a protocol based on [71]. The quantification of the different plant growth regulators and the loss correction was made using deuterium-labeled standards: d_7_-BA (10 ng); d_6_-ABA, d_5_-AIA, DHJA, d_3_-DHZ, d_6_-SA (20 ng); d_5_-BK y d_2_-GA1 (40 ng). Samples were re-suspended in 150 μL of 100 % methanol and filtered through a 0.2 μm, 4 mm regenerated cellulose filter (Captiva, Agilent, CA, USA). All compounds were separated out by ultra-high performance liquid chromatography (UHPLC, 1290 Agilent Technologies, CA, USA) and quantification of all plant growth regulators (PGRs) analyzed was conducted in a Triple Quad (LC/MS 6460, Agilent Technologies, CA, USA) using the protocol described in [72] for cytokinins. Chromatographic separation was made using a reverse phase precolumn + column (ZorbaxEclipse Plus C18- 2.1 × 5 mm, 1.8 μm and Zorbax Eclipse Plus C18 RRHD 2.1 × 50 mm, 1.8 μm, Agilent Technologies, CA, USA) kept at 40 °C.

As mobile phase two solvents, MeOH and ultra-purified water were used, both buffered at pH4 with ammonium formate (10 mM). A linear gradient of MeOH from 10–50 % in 7 min, then 100 % maintained for 2 min was used for analyte elution at a flow rate of 0.45 mL/min. Plant growth regulators were quantified by dynamic multireaction monitoring (MRM) of their [M + H] + and the appropriate product ions, using optimized cone voltages and collision energies for the diagnosis of each PGR analyzed. Data acquisition and processing were performed using Masshunter Workstation software B 04.00 (Agilent technologies, CA, USA).

Boxplots and Student’s *t* test were made using R environment (http://www.R-project.org). Differences were considered significant with a *P-value <* 0.05.

### Availability of supporting data

All the supporting data of this article are included as Additional files.

## Additional files

Additional file 1: Figure S1. Phenotypes of Leiria and Tamrabta plantlets. Tammrabta in the left side and Leiria in the right side. **Figure S2.** Location of the provenances, Leiria and Tamrabta. Map was adapted from the European Forest Genetic Resources Programme (EUFORGEN, http://www.euforgen.org/). **Figure S3.** Multidimensional scaling (MDS) plot for microarray samples. **Figure S4.** KEGG metabolism overview map. **Note S1.** Limma R script. **Note S2.** EdgeR R script. **Note S3.** Gene dataset used in the KAAS software. (PDF 907 kb) Additional file 2: Table S1. Limma DE gene table of the spots with foreground different to background. **Table S2.** EdgeR DE gene table. **Table S3.** Comparison between PINARRA3 and RNA-Seq data. **Table S4.** Mapman categories from DE genes. **Table S5.** Contig ID for Reference transcriptome. **Table S6.** Primers used for qPCR. (XLSX 4413 kb)

## Abbreviations

RNA-Seq: RNA sequencing
NCBI: National Center for Biotechnology Information
GEO: NCBI’s Gene Expression Omnibus
RIN: RNA integrity number
CNAG: Centro Nacional de Análisis Genómico
UHPLC: Ultra-high performance liquid chromatography
LC/MS: Liquid chromatography–mass spectrometry
MRM: Dynamic multireaction monitoring
MeOH: Methanol
logFC: Fold change logarithm
FDR: False discovery rate
ABA: Abscisic acid
GAs: Gibberellins
JA: Jasmonic acid
SA: Salicylic acid
AQP: Aquaporin
DE: Differentially expressed
