# Supplementary material for: Transcriptome-wide analysis supports environmental adaptations of two Pinus pinaster populations from contrasting habitats
Source: BMC Genomics. 2015 Nov 6;16:909. doi: 10.1186/s12864-015-2177-x (PMC4636790; doi:10.1186/s12864-015-2177-x)
Supplement: Additional file 1: Figure S1. — Phenotypes of Leiria and Tamrabta plantlets. Tammrabta in the left side and Leiria in the right side. Figure S2. Location of the provenances, Leiria and Tamrabta. Map was adapted from the European Forest Genetic Resources Programme (EUFORGEN, http://www.euforgen.org/). Figure S3. Multidimensional scaling (MDS) plot for microarray samples. Figure S4. KEGG metabolism overview map. Note S1. Limma R script. Note S2. EdgeR R script. Note S3. Gene dataset used in the KAAS software. (PDF 907 kb) [file 12864_2015_2177_MOESM1_ESM.pdf]

Figure S1.

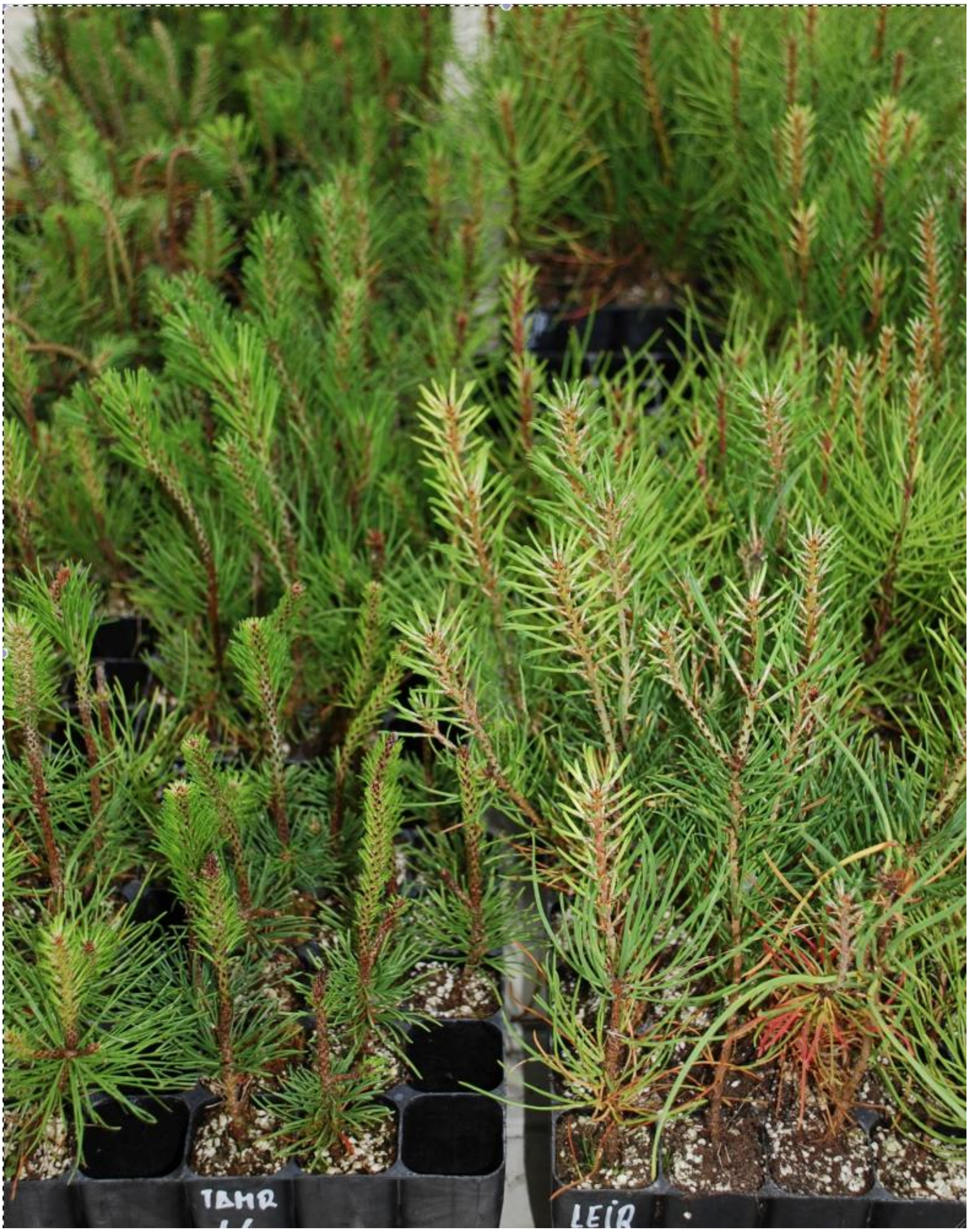

Figure S1. Phenotypes of Leiria and Tamrabta plantlets. Tammrabta in the left side and Leiria in the right side.

Figure S2.

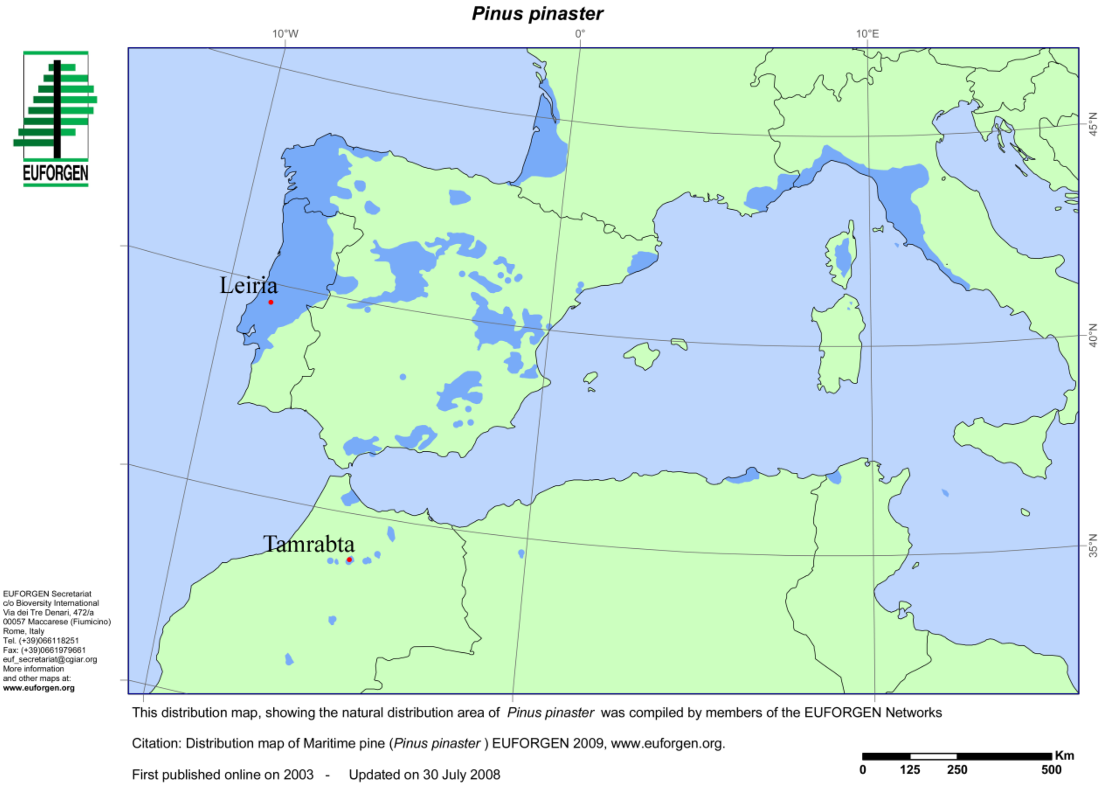

Figure S2. Location of the provenances, Leiria and Tamrabta. Map was adapted from the European Forest Genetic Resources Programme (EUFORGEN, http://www.euforgen.org/).

Figure S4.

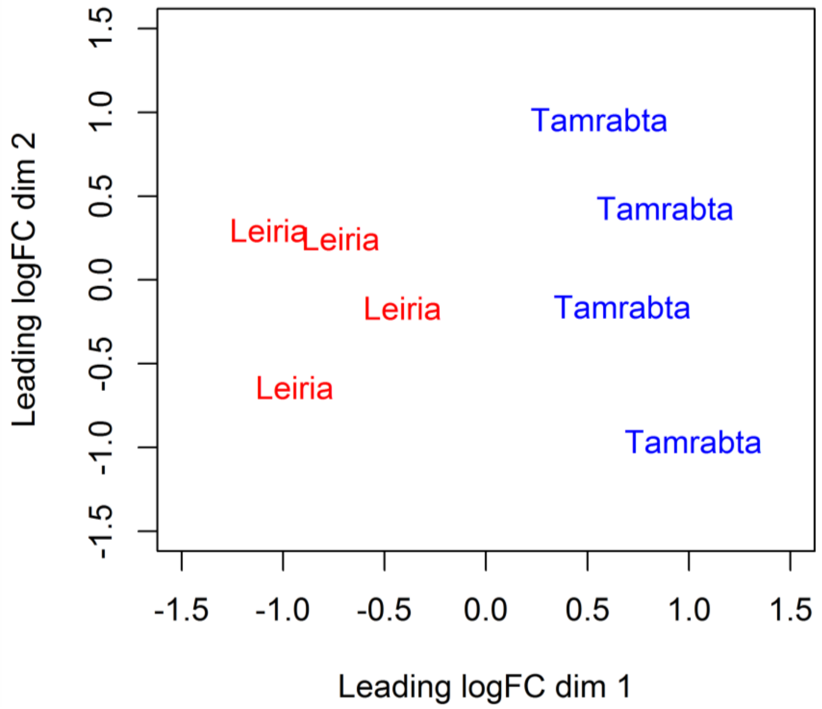

Figure S3. Multidimensional scaling (MDS) plot for microarray samples.

Figure S4.

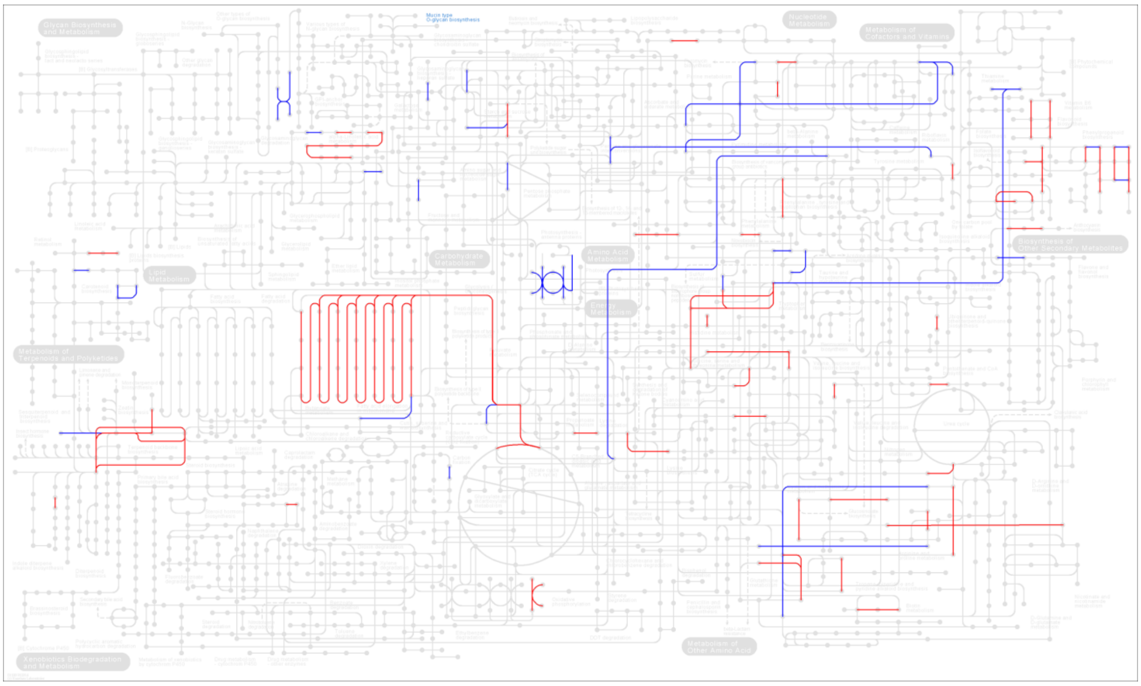

Figure S3. KEGG metabolism overview map.

Note S1. Limma R script.

```
getwd();
workingDir = "C:/Users/...";
setwd(workingDir);
library(limma)
library(RColorBrewer)
#Data quality test
myfun <- function(x, threshold=50) {
okgreen <- abs(x[,"F532 Median"]-x[,"F532 Mean"]) < threshold
as.numeric(okgreen)
}
# Read the targets file
targets <- readTargets()
#Load Microarray raw data
x <- read.maimages(targets$FileName,source="genepix",green.only=TRUE)
#Data annotation
x$genes <- readGAL()
names(x$genes)
#Array layout load
x$printer <- getLayout(x$genes)
names(x$printer)
#Background correction
y <- backgroundCorrect(x,method="normexp", offset = 50)
#Quantile normalization
y <- normalizeBetweenArrays(y,method="quantile")
#Now filter out control probes and low expressed probes. To get an idea of how bright
#expression probes should be, we compute the 95% percentile of the negative control probes
#on each array. We keep probes that are at least 10% brighter than the negative controls on
#at least three arrays (because there are three replicates): La GAL tiene valores 1, 0 y -1 en la
columna ControlType.
neg95 <- apply(y$E[y$genes$ControlType==-1,],2,function(x) quantile(x,p=0.95))
cutoff <- matrix(1.1*neg95,nrow(y),ncol(y),byrow=TRUE)
isexpr <- rowSums(y$E > cutoff) >= 3
table(isexpr)
#Regular probes are code as \0" in the ControlType column.
y0 <- y[y$genes$ControlType==0 & isexpr,]
#DEG analysis
Treatment <- targets[,"Cy3"]
levels <- c("Tamrabta","Leiria")
Treatment <- factor(Treatment,levels=levels)
design <- model.matrix(~0+Treatment)
colnames(design) <- c("Tamrabta","Leiria")
fit <- lmFit(y0,design)
contrast.matrix <- makeContrasts(Leiria-Tamrabta, levels=design)
fit2 <- contrasts.fit(fit, contrast.matrix)
fit2 <- eBayes(fit2)
```

Note S2. EdgeR R script.

```
getwd();
workingDir = "C:/Users...";
setwd(workingDir);
library(limma)
library(edgeR)
#Read the targets file
targets <- readTargets()
#Load read count data
x <- read.delim(file="ReadCount_data.txt",row.names="id",stringsAsFactors=FALSE)
y <- DGEList(counts=x,group=targets$Treatment)
colnames(y) <- targets$Label
#Gene filtering by read (at least 1 read into two samples)
keep <- rowSums(cpm(y)>1) >= 2
y <- y[keep,]
#Normalization factors
y <- calcNormFactors(y)
#Sample dispersion
y <- estimateCommonDisp(y, verbose=TRUE)
y <- estimateTagwiseDisp(y)
#Normalization (CPM)
norm <- cpm(y)
lognorm <- cpm(y,log=TRUE)
#Differential expression analysis
et <- exactTest(y)
#Results saving
write.table(et$table, file = "DEGeneList.txt")
top <- topTags(et, n=200000)
write.table(top, file = "TOPDEGeneList.txt")
topcpm <- cpm(y)[rownames(top), ]
write.table(topcpm, file = "CPMTOPDEGeneList.txt")
suma <- summary(de <- decideTestsDGE(et))
write.table(de, file = "decideTestsDGE.txt")
```

Note S3. Gene dataset used in the KAAS software.

hsa, dme, cel, ath, sce, cho, eco, nme, hpy, rpr, bsu, lla, cac, mge, mtu, ctr, bbu, syn, bth, dra, aae, mja, ape, osa, vvi, olu, cme
